# Ultrasonic Thickness Measurement Method and System Implementation Based on Sampling Reconstruction and Phase Feature Extraction

**DOI:** 10.3390/s23229072

**Published:** 2023-11-09

**Authors:** Wenqiang Gong, Xuanze Wang, Zhenyu Yang, Zhongsheng Zhai, Wei Feng, Da Liu

**Affiliations:** Hubei Key Laboratory of Modern Manufacturing Quantity Engineering, College of Mechanical Engineering, Hubei University of Technology, Wuhan 430068, China

**Keywords:** ultrasonic signal, sampling reconstruction, moving sine fitting, multiplier

## Abstract

The existing ultrasonic thickness measurement systems require high sampling frequencies for echo signal acquisition, leading to complex circuit designs and high costs. Moreover, extracting the characteristics of ultrasonic echo signals for accurate thickness measurement poses significant challenges. To address these issues, this paper proposes a method that utilizes conventional sampling frequencies to acquire high-frequency ultrasonic echo signals, overcoming the limitations of high-frequency data acquisition imposed by the Nyquist–Shannon sampling theorem. By employing an improved sampling reconstruction technique, the multi-cycle sampling signals are reconstructed and rearranged within a single cycle, effectively increasing the equivalent sampling frequency. Additionally, a combination of coarse estimation using fast Fourier transform (FFT) and precise phase extraction using the moving sine fitting algorithm is proposed for accurate thickness measurement, resolving the limitations of common thickness measurement methods such as peak detection, envelope detection, and Hilbert autocorrelation in terms of low measurement accuracy. Experimental results obtained from thickness measurements on 45 steel ultrasonic test blocks within the range of 3 mm to 20 mm indicate a measurement error of ±0.01 mm, while for thicknesses ranging from 1 mm to 50 mm, the measurement error is ±0.05 mm.

## 1. Introduction

Ultrasonic penetration ability, applied to metal thickness measurement [1], has the characteristics of low cost, fast speed, and high accuracy [2]. For the aerospace and nuclear industries and other harsh environments or high-risk areas in the use of pressure vessels [3], process equipment, and other structures and components, the use of ultrasonic wall thickness measurement is timely and feasible [4,5]. It provides an effective basis for the components of the operational reliability of the judgment in the service life [6,7].

The ultrasonic frequency is high, and general thickness measurement using the ultrasonic probe is between 0.5 and 15 MHz [8]. The current ultrasonic thickness measurement system requires a specialized high-speed AD sampling chip and FPGA controller [9]. It makes the system complex and adds unnecessary burden and cost. In addition, the accuracy of ultrasonic thickness measurement relies on the time of flight (TOF) [10] in ultrasonic thickness measurement, i.e., the accuracy of ultrasonic echo signal period extraction. The simplest way to determine the TOF is to set a fixed threshold, the time difference between the moment when the echo signal amplitude exceeds the threshold for the first time and the initial moment of the excitation signal. It is simple to compute but affected by the size of the set threshold; the estimation error is large and cannot be modeled for analysis.

Kim Y H utilizes the peak method for thickness measurement [11]. The peak method searches for the maximum peak point of the echo signal in all time domains. Calculating the distance between two neighboring peaks. The corresponding time is the TOF. This method is susceptible to noise and external interference, resulting in a shift of the maximum peak searched for, a large measurement error, and a decrease in accuracy. The literature [12] proposed the envelope peak method. The echo signal envelope fitting is processed through the measurement of the neighboring envelope peak distance to derive the TOF. Avoiding the impact of noise on the measurement accuracy, but there is the disadvantage that searching for the peak position is not permitted when the amplitude of the envelope signal is close to the shortcomings. Zhou Qianqian [13] proposed the Hilbert autocorrelation algorithm to process the echo signal, using the Hilbert transform to extract the features and then autocorrelation to find the TOF to improve the measurement accuracy.

In view of the above two problems, this paper proposes an ultrasonic thickness measurement processing method with sample reconstruction and FFT rough estimation [14]. Using the FFT algorithm to calculate the peak point of amplitude-frequency characteristics, the frequency of the sampled signal can be roughly estimated. The phase is accurately extracted by the moving sine fitting algorithm. The purpose of ultrasonic thickness measurement can be realized by using only the low-frequency AD sampling function inside the STM32F407 mi-processor (STMicroelectronics, Switzerland) and the developed moving sine fitting algorithm. The limitations of high-frequency sampling required to acquire ultrasonic echo signals and the defects of low accuracy and poor stability of the traditional direct calculation of workpiece thickness by peak point position characteristics are solved. The cost-effectiveness of the ultrasonic thickness measurement system is demonstrated.

## 2. Improved Sampling Reconstruction Technique

Sampling reconstruction can generally be divided into two sampling methods: sequential sampling reconstruction [15] and random sampling reconstruction [16]. The ultrasonic thickness measurement system sampling method using improved sampling reconstruction technology involves setting the sampling frequency and excitation frequency, determining a fixed number of excitation cycles and the total number of sampling points to achieve continuous acquisition of sampling points, and reordering the sampling points to complete the signal reconstruction. To meet the purpose of the improved sampling reconstruction technique, the excitation frequency $f_{e}$ of the ultrasound probe and the sampling frequency $f_{s}$ of the ultrasound signal should meet the following requirements:

(1) $$\frac{n}{f_{s}}=\frac{m}{f_{x}}⟹nT_{s}=mT_{x}$$

where *n* and *m* are prime numbers of each other, *n* is the total number of sampling points, *m* is the number of ultrasonic excitation signal cycles, $T_{s}$ is the sampling period of the signal, and $T_{x}$ is the excitation signal period. Consequently, it is necessary to ensure that the total time of the *n* sampling cycles is equal to the total time of the *m* excitation signal cycles. The sampling period of the improved sampling reconstruction technique is much smaller than the excitation period, and the sampling period of the sequential sampling reconstruction is the sum of the excitation cycle time and the step increment delay time, which is set to be larger than the excitation cycle time. The signal reconstruction is accomplished by synthesizing *n* points sorted into one signal cycle through the reconstruction sorting algorithm, in which the important parameters *m*, *n*, $T_{s}$, and $T_{x}$ need to be set according to the system clock of the STM32F407 microprocessor following the improved sample reconstruction formula. The sampling reconstruction frequency $f_{se}$ is shown in Equation (2).

(2) $$f_{se}=nf_{x}=mf_{s}$$

**Figure 1:**
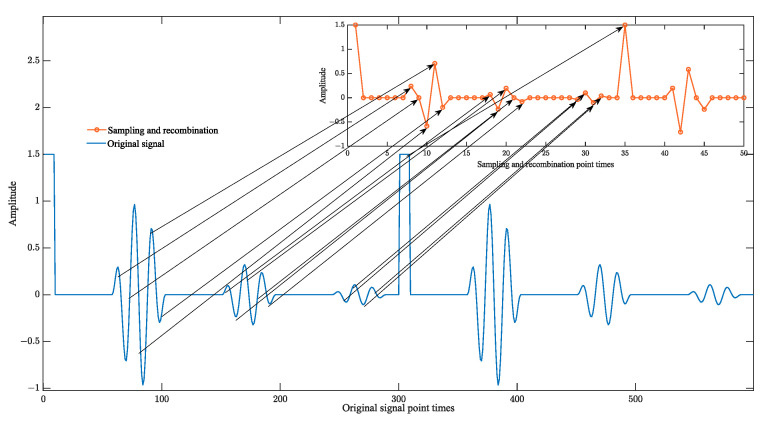
Schematic diagram of the principle of improved sampling reconstruction.

Compared with the original ultrasound signal sampling frequency, sampling reconstruction frequency increases by a factor of m. It can meet the requirements of periodic high-frequency signal sampling. The principle of the improved sampling reconstruction is shown in Figure 1.

In the above figure, each ultrasonic excitation cycle contains multiple gradually attenuated ultrasonic echo signals. The thickness measurement system in the STM32F407 microprocessor set parameters *m*, *n*, $T_{s}$, $T_{x}$ should meet the improved sampling reconfiguration of Equation (1), so *m* excitation cycle contains exactly *n* sampling points, and m and n reciprocity to ensure that *n* sampling points are not duplicated to be uniformly inserted into a signal excitation cycle can be synthesized into a complete high-frequency signal cycle.

**Figure 2:**
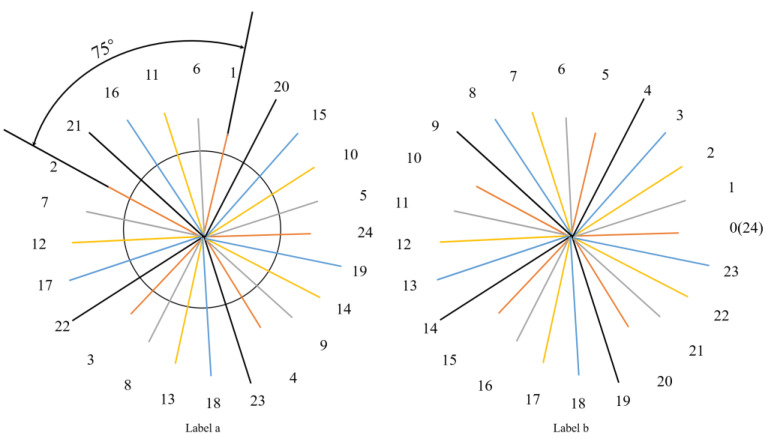
(**a**) Raw sampling data sequence; (**b**) rearrangement of sampled data series.

Figure 2, for example, shows the basic principle of the signal reconstruction algorithm, assuming that the sampling points are collected once every 75° interval, the original periodic signal is one cycle for every 360° interval, the total number of sampling points is 24, and the number of signal cycles is 5, which meets the requirements of the improved sampling reconstruction formula, and the sampling points can be synthesized completely into one cycle, but the sequence of sampling points is not sequential, and the sampling points are needed to be synthesized into one cycle. However, the sequence of sample points is not sequential, and it is necessary to sort the sample points synthesized into a signal cycle. The signal reconstruction schematic is shown in Figure 2.

The transformation of the original sampled data sequence to the rearranged sampled data sequence in the figure is obtained according to the reconstruction algorithm, whose specific derivation formula is shown in (3):

(3) $$\left\{\begin{array}{c} r^{*}=rm-\left[\frac{rm}{n}\right]\cdot n\\ y\left(r^{*}\right)=x\left(r\right) \end{array}\right.$$

where $r^{*}$ is the rearrangement sequence number of the signal, *r* is the sequence number of the sampled signal, from 0 to *n* − 1 sequential sequence. *m* is the number of signal cycles, *n* is the total number of sampling points, [] is the sign of downward taking an integer, the rearrangement sequence signal is $y\left(r^{*}\right)$, and the sampling sequence signal is $x\left(r\right)$.

**Figure 3:**
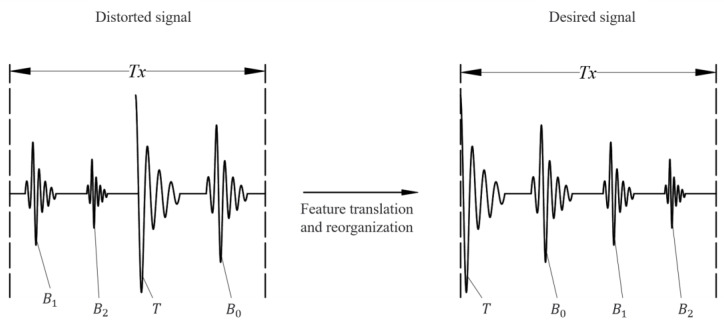
Schematic diagram of ultrasound echo signal recovery.

Due to the jitter in the sampling and excitation clocks of the microprocessor [17], it is difficult to ensure that the initial position of the sampling clock is synchronized with the initial position of the excitation signal. The position of the initial sampling point is random, and the reconstructed signal will be distorted. As shown in Figure 3, the maximum point is found based on the echo peak feature. The distorted signal is recombined by feature-point translation. The ultrasonic echo signal characteristics within the signal period are effectively recovered. The accuracy of the ultrasonic echo signal is guaranteed.

In the figure, *T* is the ultrasonic emission wave, and $B_{0}$, $B_{1}$, and $B_{2}$ are the first ultrasonic echo, the second ultrasonic echo, and the third ultrasonic echo in the ultrasonic echo signal, respectively.

Through the above analysis, the effect of fast sampling can be achieved by simply setting the relative sampling frequency and excitation frequency, as well as determining the number of sampling points and the number of signal cycles. Compared with the traditional sampling reconstruction method, the sampling efficiency is higher and faster.

## 3. Thickness Measurement System Hardware Design

### 3.1. Hardware System Design

**Figure 4:**
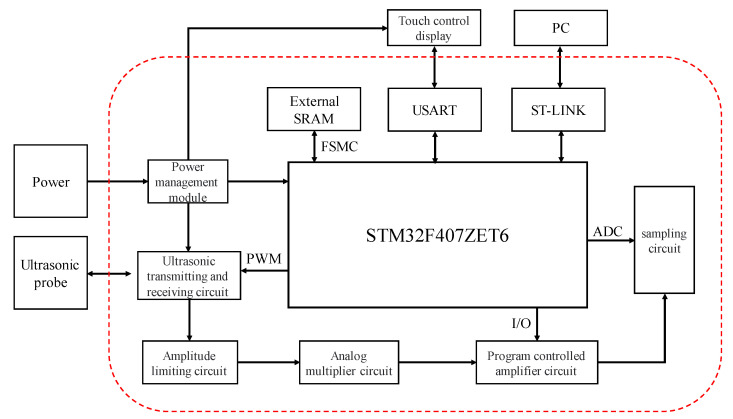
Overall block diagram of the hardware circuit.

**Figure 5:**
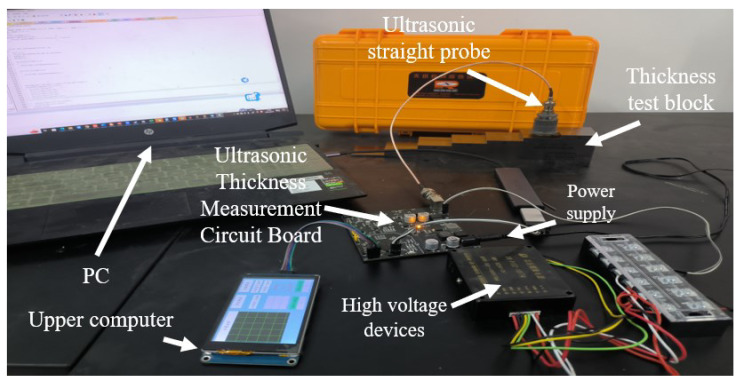
The actual circuit diagram of the experiment.

The overall design of the hardware circuit for ultrasonic thickness measurement is shown in Figure 4. The actual circuit diagram of the experiment is shown in Figure 5. It mainly includes the STM32F407 minimum system circuit, external SRAM circuit, power supply circuit, ultrasonic transmitter–receiver circuit, limiter circuit, analog multiplier circuit, programmable amplifier circuit, sampling circuit, and touch display circuit.

Among them, the STM32F407 minimum system is the core of the thickness measurement system. It is mainly responsible for running the program, realizing the excitation pulse (PWM pulse) generation, and collecting and storing the echo signal. Using a noise reduction algorithm and a moving sine fit phase feature extraction algorithm to measure the thickness of the object. And then transmitting the measured results to a touchscreen display via the USART communication protocol. The external SRAM circuit is mainly used to expand the memory for the programmed embedding of the wavelet threshold noise reduction algorithm, FFT, and moving sine fitting algorithm. The power management module is mainly responsible for managing the system voltage distribution. The output 400 V DC voltage is used as the excitation voltage of the ultrasound probe. Output 5 V is the working voltage of the touch panel display. Output 3.3 V is the working voltage and reference voltage of the STM32F407 microprocessor. The ultrasound transmitter–receiver circuit is used to control the ultrasound probe operation as well as the ultrasound signal transmitting and receiving. The limiter circuit is to reduce the amplitude of the received echo signal to protect the circuit. The analog multiplier circuit is the ultrasonic echo signal self-multiplication process, the alternating ultrasonic signals into pulsating DC signals conducive to echo signal acquisition. The programmable amplifier circuit amplifies the weak echo signals, the sampling circuit samples and reconstructs the echo signals, and the touch panel display facilitates man-machine operation, the issuance of commands, and the direct display of measurement results. 

### 3.2. Analog Multiplier Circuit

The analog multiplier circuit [18] mainly consists of the multiplier chip AD835 and its peripheral circuits to self-multiply the ultrasound echo signal. The alternating ultrasound signal into a pulsating DC signal is the main role of two points. The first is to self-multiply the ultrasonic echo signal to provide a basis for the subsequent FFT rough estimation algorithm and reduce the arithmetic pressure of the main control chip. The second is that the sampling amplitude of the echo signal can be increased to facilitate signal sampling.

**Figure 6:**
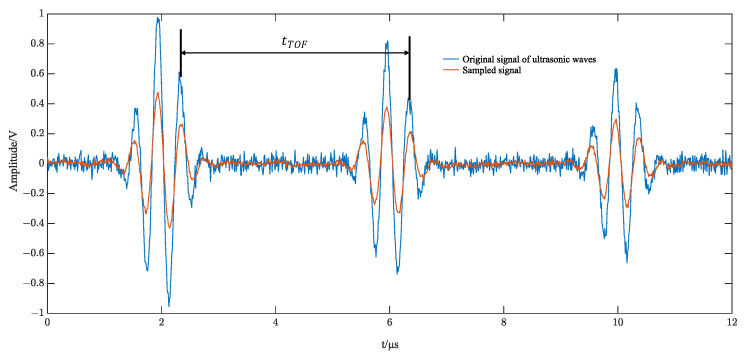
Raw signal sampling simulation.

**Figure 7:**
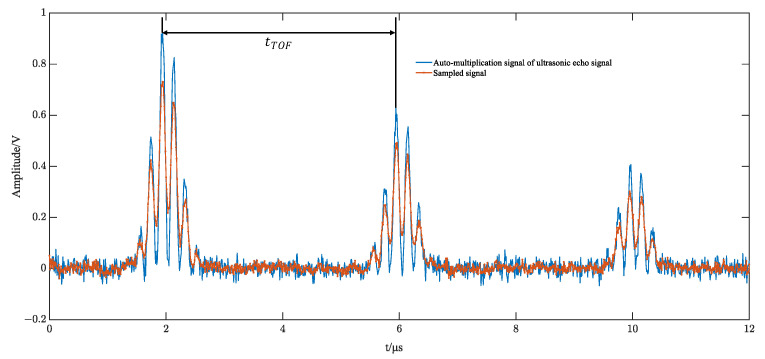
Simulation of self-multiplying echo signal sampling.

Low-frequency samplers have the nature of a smoothing filter. Direct sampling of the original alternating high-frequency ultrasound echo signal results in a sampled signal with amplitude attenuation. Using ultrasonic echo signal modeling to create simulation comparisons, the sampling amplitude of the original signal and the self-multiplying sampled signal changes, as shown in Figure 6 and Figure 7.

## 4. System Software Design and Implementation

### 4.1. System Software Design Process

**Figure 8:**
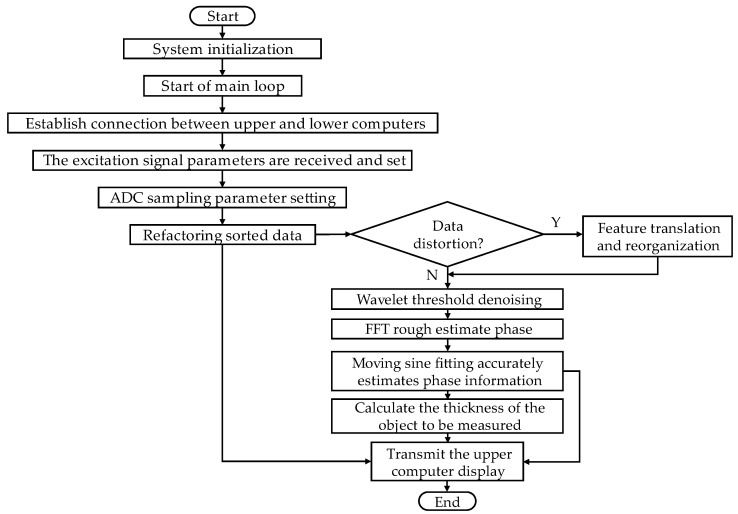
Thickness measurement system program flow chart.

The system software design flow is shown in Figure 8. In the thickness measurement system, the host computer is set up to meet the sampling reconstruction requirements of the excitation frequency $f_{x}$ and sampling frequency $f_{s}$ and other parameters, sent to the lower computer STM32F407, to control the lower computer to produce excitation signals for sampling reconstruction and noise cancellation processing, the use of moving sine fitting algorithms to extract the phase characteristics of the signal, and derive the workpiece under test TOF. Based on the calibrated speed of sound, the thickness of the workpiece is calculated, and the result is transmitted to the touch-screen display of the host computer for display.

### 4.2. Wavelet Noise Reduction Method for Ultrasound Echo Signals

In the ultrasonic thickness measurement process, the system power supply perturbation and external interference will introduce noise to the ultrasonic echo signal [19]. To improve the accuracy of ultrasonic thickness measurement and reduce the impact of noise on the ultrasonic echo signal, there needs to be noise reduction on the echo signal. General noise reduction methods include hardware noise reduction [20] and digital processing noise reduction [21]. Hardware noise reduction for the device performance requirements is high, and the cost is expensive. At this stage, digital signal processing echo noise reduction technology has matured, and the wavelet noise reduction method [22] has a good noise reduction effect and is widely used.

**Figure 9:**
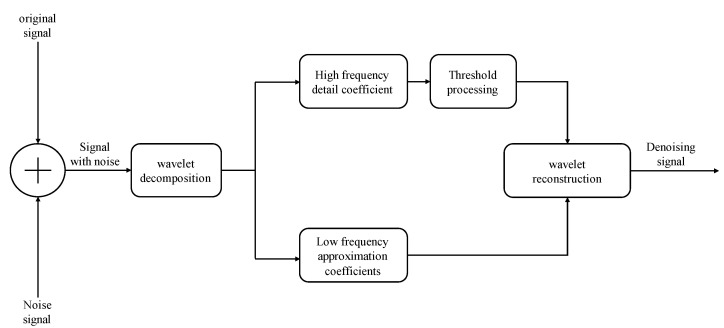
Schematic diagram of the principle of wavelet thresholding noise reduction method.

The noise-containing signal is wavelet decomposed into a low-frequency approximation part and a high-frequency detail part. The useful signal is located in the low-frequency part. The noise signal is located in the high-frequency detail part. The wavelet-decomposed high-frequency detail coefficients are subjected to threshold quantization and wavelet reconstruction with the low-frequency approximation coefficients to realize wavelet threshold noise reduction, the principle of which is shown in Figure 9.

According to the characteristics of the ultrasonic echo signal in this paper, the dbN orthogonal wavelet is selected as the wavelet function in the threshold noise reduction algorithm. The selection of the threshold value in the wavelet threshold noise reduction method plays a crucial role. If the threshold is too large, the mixed noise cannot be eliminated, and the noise reduction effect is poor. There are several commonly used thresholding methods.

Fixed threshold (Sqtwolog rule) with the threshold derivation formula shown in (4).

(4) $$\left\{\begin{array}{l} \lambda_{s}=\sigma_{0}\sqrt{2\ln\left(N\right)}\\ \sigma_{0}=\frac{med\left(\left|d_{1,k}\right|\right)}{0.6745} \end{array}\right.$$

where $\sigma_{0}$ is the noise standard deviation, *N* is the signal length, *med* is the median, and $d_{1,k}$ is the sequence of wavelet coefficients in the first layer.

The unbiased risk estimation threshold (Rigrsure rule), based on the principle of unbiased likelihood estimation, has a threshold derivation formula shown in (5).

(5) $$\lambda_{r}=\sigma_{0}\sqrt{min\left\{\frac{N-2k+\sum {r_{1,k}}^{2}+\left(N-k\right){r_{1,k}}^{2}}{N}\right\}}\left(k=1,2,3,\ldots ,N\right)$$

where $r_{1,k}$ is the ascending order of the wavelet coefficients of the first layer.

The maximum–minimum criterion threshold (Minimaxi rule) is selected concerning the noise standard deviation, and its threshold derivation formula is shown in (6).

(6) $$\lambda_{m}=\left\{\begin{array}{l} \sigma_{0}\left(0.3936+0.1829\;\log_{2}N\right)\;\;\;\;\;\;\;\;\;\;\;\;\;\;\;\;\;\;\;\;\;\;\;\;\;\;\;\;\;N>32\\ 0\;\;\;\;\;\;\;\;\;\;\;\;\;\;\;\;\;\;\;\;\;\;\;\;\;\;\;\;\;\;\;\;\;\;\;\;\;\;\;\;\;\;\;\;\;\;\;\;\;\;\;\;\;\;\;\;\;\;\;\;\;\;\;\;\;\;\;\;\;\;\;\;\;\;\;N\leq 32 \end{array}\right.$$

The heuristic threshold (Heursure rule), which is the preferred choice between a fixed threshold and an unbiased risk estimation threshold, has a threshold derivation formula shown in (7).

(7) $$\lambda_{n}=\left\{\begin{array}{c} \lambda_{s}\;\;\;\;\;\;\;\;\;\;\;\;\;\;\;\;\;\;\;\;\;\;\;\;\;\;\;\;\;\;\;\;\;\;\;\;\;\;\;\;\;\;\;\;\;\;\;\;\;\frac{\sum d_{1,k}}{N}<\log_{2}N\sqrt[{\frac{3}{2}}]{N}\\ \min\left(\lambda_{s},\lambda_{r}\right)\;\;\;\;\;\;\;\;\;\;\;\;\;\;\;\;\;\;\;\;\;\;\;\;\;\;\;\;\;\;\;\;\;\frac{\sum d_{1,k}}{N}<\log_{2}N\sqrt[{\frac{3}{2}}]{N} \end{array}\right.$$

Multi-resolution thresholding is an improvement over fixed thresholding. The wavelet decomposition process, wavelet coefficients, and noise signals are affected by the number of decomposition layers, and the threshold derivation formula is shown in (8).

(8) $$\left\{\begin{array}{c} \lambda_{i}=\sigma \frac{\sqrt{2\ln length\left(d_{i,k}\right)}}{\ln\left(i+1\right)}\\ \sigma =\frac{med\left(\left|d_{i,k}\right|\right)}{0.6754} \end{array}\right.$$

where i is the number of decomposition layers, length is the length, and $d_{1,k}$ is the sequence of wavelet coefficients in the ith layer.

Based on the root-mean-square error (RMSE) and signal-to-noise ratio (SNR) of the noise-canceled signal as an evaluation criterion, the equations are shown in (9) and (10).

(9) $$RMSE=\sqrt{\frac{\sum {\left[y\left(n\right)-{y\left(n\right)}^{*}\right]}^{2}}{N}}$$

(10) $$SNR=10\mathrm{lg}\frac{\sum {y\left(n\right)}^{2}}{\sum {\left[y\left(n\right)-{y\left(n\right)}^{*}\right]}^{2}}$$

The smaller the root-mean-square error (RMSE) of the noise-canceled signal, the better the noise-canceling effect; the larger the signal-to-noise ratio (SNR), the better the noise-canceling effect; and vice versa.

**Figure 10:**
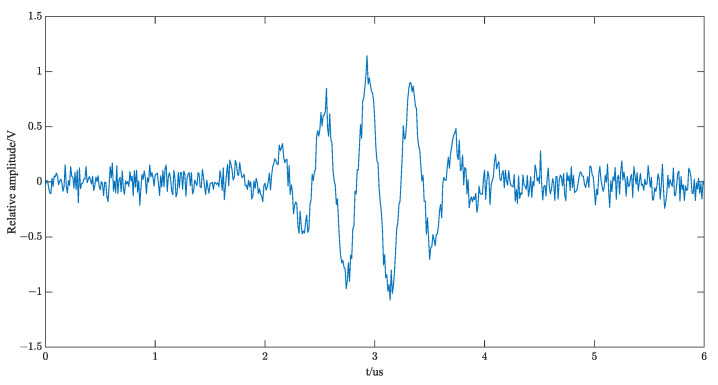
Single echo signal with SNR = 10 dB noise.

In the actual ultrasonic thickness measurement, the ultrasonic echo signal is mixed with noise. To simulate the actual echo signal, random Gaussian white noise is added to the ideal single echo signal, and the model of the simulated mixed-noise echo signal is shown in Equation (11).

(11) $$y\left(t\right)=s\left(\theta ;t\right)+v\left(t\right)$$

where $s\left(\theta ;t\right)$ is the single echo and v(t) is the Gaussian white noise, and the specific echo of Gaussian white noise with 10 dB added is shown in Figure 10.

The above single-echo signal model can be deduced from the multiple-echo signal; each time the reflected wave is received, it is gradually attenuated, and the amplitude is getting smaller and smaller. Considering this layer of the problem, the multiple-echo model is shown in Equation (12).

(12) $$y_{r}\left(t\right)=\sum_{m=1}^{M}s\left(\theta ;t\right)+v\left(t\right)$$

where *M* is the number of echo signals and $s\left(\theta ;t\right)$ is the mth echo waveform.

**Figure 11:**
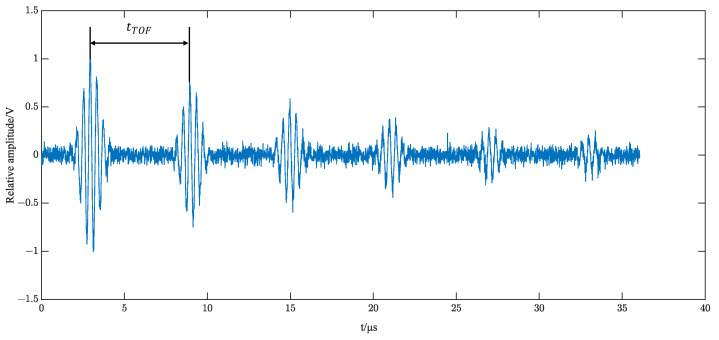
Multiple echo signal with SNR = 10 dB noise.

Keeping the above single-echo eigenvector parameters unchanged, setting the attenuation factor to 0.6 and the time interval =4 μs, the simulated ultrasound echo signal is shown in Figure 11.

**Figure 12:**
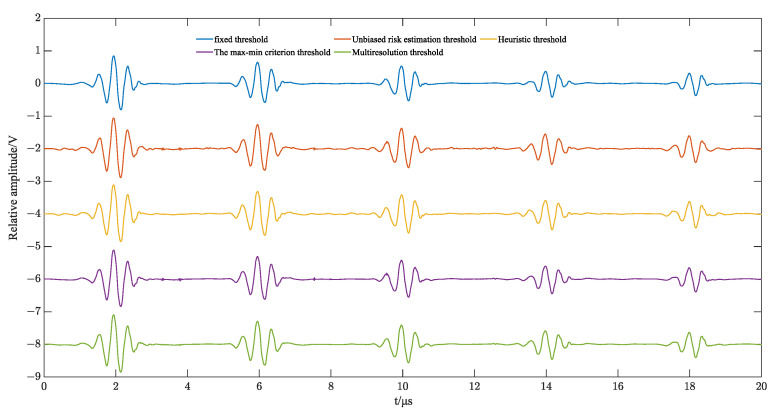
Simulation effect of different thresholding methods.

Based on [23], wavelet 5-layer decomposition is chosen to simulate the above five thresholding methods, and the simulation results are shown in Figure 12. The root mean square error (RMSE) and signal-to-noise ratio (SNR) are calculated, and the results are shown in Table 1.

According to Table 1, it can be seen that the multi-resolution thresholding method has the smallest root-mean-square error (RMSE) and the largest signal-to-noise ratio (SNR), and the signal noise reduction effect is better than other thresholding methods. Therefore, multiresolution thresholding as the wavelet threshold was selected.

### 4.3. Algorithm for Feature Extraction of Echo Signals

After noise reduction of the echo signal using wavelet multiresolution thresholding, the signal has a high signal-to-noise ratio, and this paper proposes an algorithm to accurately extract the phase information of the echo signal. Extracting the phase information via FFT rough estimation of the signal and shifted sine fitting. Then TOF is calculated from the phase difference of the feature positions.

#### 4.3.1. FFT Rough Estimation Algorithm

The fast Fourier transform (FFT) algorithm is commonly used for spectral simulation and analysis of signals, using a time-to-frequency domain signal processing method to obtain a discrete sequence of equally spaced samples containing frequency components [24]. To extract the frequency components of the low-frequency part of the ultrasonic echo signal, the direct FFT processing of the ultrasonic echo signal cannot determine the location of the low-frequency frequency components, while the self-multiplied ultrasonic echo signal can be decomposed to clarify the location of the low-frequency, and the decomposition of the specific self-multiplied equivalent ultrasonic echo modeling decomposition formula is shown in (17).

(13) $$X\left(t\right)=f^{2}\left(t\right)={f_{m}}^{2}\left(\varphi \right)\cdot \cos^{2}\left(2\pi f_{l}t\right)\cdot \cos^{2}\left(2\pi f_{h}t\right)\cdot f^{2}\left(\alpha \right)$$

Since $f_{m}\left(\varphi \right)$ and $f\left(\alpha \right)$ are pulse constant signals and attenuation factors, it can be simplified as:

(14) $$\begin{array}{ll} X^{*}(t) & =\cos^{2}\left(2\pi f_{l}t\right)\cdot \cos^{2}\left(2\pi f_{h}t\right)\\ & =\frac{1+\cos(4\pi f_{l}t)}{2}\cdot \frac{1+\cos(4\pi f_{h}t)}{2}\\ & =\frac{1}{4}+\frac{\cos(4\pi f_{l}t)}{4}+\frac{\cos(4\pi f_{h}t)}{4}+\frac{\cos(4\pi f_{l}t)\cdot \cos(4\pi f_{h}t)}{4}\\ & =\frac{1}{4}+\frac{\cos(4\pi f_{l}t)}{4}+\frac{\cos(4\pi f_{h}t)}{4}+\frac{\cos(4\pi f_{l}t-4\pi f_{h}t)}{8}+\frac{\cos(4\pi f_{l}t+4\pi f_{h}t)}{8} \end{array}$$

The low-frequency frequency $f_{l}$ in Equation (14) is in the second sequence in the decomposition equation. Thus, the FFT transform of the self-multiplying ultrasound echo signal determines that the low-frequency spectral position is at the position of the extreme value point after the constant.

**Figure 13:**
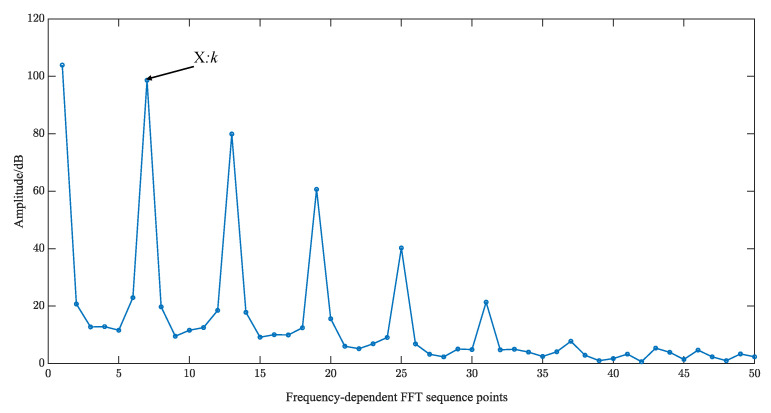
FFT spectrogram.

The self-multiplying equivalent ultrasound echo signal of length N is selected for the FFT transform, and the resulting spectrogram is shown in Figure 13.

The position of the frequency point corresponding to the low-frequency harmonics of the ultrasound echo is determined as *k* by the decomposition formula in the figure, and the formula for calculating the low-frequency frequency $f_{l}$ of the FFT is deduced based on the position of this frequency point as shown in (19).

(15) $$f_{l}=k\cdot \frac{f_{se}}{N}$$

In the formula, $f_{se}$ is the sampling reconstruction frequency, and *N* is the number of sampling points. From the above formula, it can be seen that the accuracy of low-frequency frequency calculation is affected by the sampling frequency and the number of sampling points. In the case of a certain sampling frequency, it can only increase the length of the window and deal with a large number of sampling points, which is not high in real-time, and the accuracy is poor when the FFT algorithm is used directly to count the low-frequency frequency. Therefore, in this paper, the moving sine fitting is used to accurately calculate the echo characteristic phase information and then accurately calculate the low-frequency frequency and period.

Since the moving sine fitting algorithm is used, the phase interval of the fitted sequence needs to be known. Therefore, the FFT algorithm was used to roughly estimate the ultrasound echo low-frequency harmonics, and the phase interval δ was calculated based on the echo low-frequency harmonics, as shown in (20).

(16) $$\delta =\frac{2\pi k}{N}$$

#### 4.3.2. Calculation of the Wrap Phase of a Moving Sine Fit

A segment-by-segment shifted sinusoidal fit yields information on the magnitude and phase of points in the sampled sequence of points, except for one cycle of boundary loss [25]. The essence of shifted sine fitting is to obtain the amplitude and phase of the fundamental frequency harmonics at various points in the return signal. If the ultrasound echo sequence is y(i), the *q*th sequence of segment length *l* is expressed as {y(q), y(q+1),…, y(q+l−1)} and a sine fit to it yields the amplitude A(q) and the initial phase φ(q) of the segment, respectively:

(17) $$\left\{\begin{array}{c} A(q)=\sqrt{{a_{q}}^{2}+{b_{q}}^{2}}\\ \varphi (q)=atan2(b_{q},a_{q}) \end{array}\right.$$

where $a_{q}$ and $b_{q}$ are calculated as follows:

(18) $$\left[\begin{array}{c} a_{q}\\ b_{q}\\ c_{q} \end{array}\right]=Q_{0}^{-1}\left[\begin{array}{c} \sum_{i=q}^{q+l-1}y(i)\sin(i\cdot \delta )\\ \sum_{i=q}^{q+l-1}y(i)\cos(i\cdot \delta )\\ \sum_{i=q}^{q+l-1}y(i) \end{array}\right]$$

Here the matrix $Q_{0}$ is a constant symmetric matrix:

(19) $$Q_{0}=\left[\begin{array}{ccc} \sum_{i=0}^{l-1}\sin^{2}(i\cdot \delta ) & \sum_{i=0}^{l-1}\frac{1}{2}\sin(2i\cdot \delta ) & \sum_{i=0}^{l-1}\sin(i\cdot \delta )\\ \sum_{i=0}^{l-1}\frac{1}{2}\sin(2i\cdot \delta ) & \sum_{i=0}^{l-1}\cos^{2}(i\cdot \delta ) & \sum_{i=0}^{l-1}\cos(2i\cdot \delta )\\ \sum_{i=0}^{l-1}\sin(i\cdot \delta ) & \sum_{i=0}^{l-1}\cos(2i\cdot \delta ) & 1 \end{array}\right]$$

According to the characteristics of the symmetry of the sinusoidal signal period, the fitting of the whole period sampling points has a higher accuracy. Therefore, the segment length adopted for segment-by-segment shifted sinusoidal fitting should be as close as possible to the cycle length of the ultrasonic echo signal, and here the segment length of the fitting can be designed according to the interval phase calculated by Equation (16):

(20) $$L=[\frac{2\pi }{\delta }]$$

Moving sine fitting is computationally intensive and can be greatly reduced if recursion is used for the cumulative sum operation in Equation (7) [25].

Considering that the initial phase sequence ϕ(q) sought in Equation (6) is the wrapped phase, the unwrapped phase can be obtained as ϕ(q) by the unwrapping operation. A least-squares linear fit to the data set [q, ϕ(q)] is performed, i.e., the following equation is satisfied:

(21) $$\phi (q)=s_{1}q+s_{0}$$

After the straight line fitting calculating the fitted slope parameter $s_{1}$, the refined estimation of the ultrasound echo signal time difference is:

(22) $$T_{e}=\frac{2\pi \cdot T_{x}}{n\cdot s_{1}}$$

The thickness of the workpiece is:

(23) $$H=\frac{\pi T_{x}}{n\cdot s_{1}}\cdot v_{s}$$

$v_{s}$ is the velocity of ultrasonic propagation.

#### 4.3.3. Calculation of TOF

**Figure 14:**
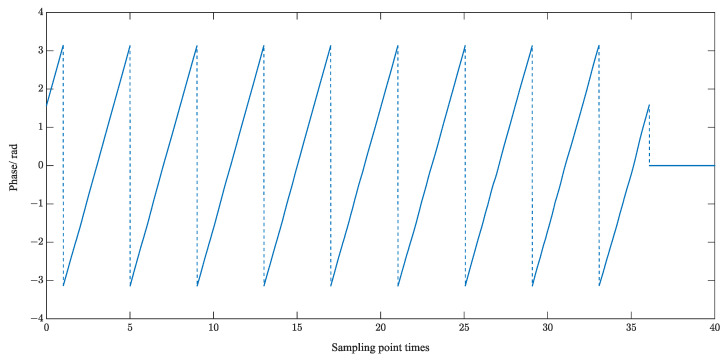
Wrapped phase diagram of the echo sampled signal.

The computer extracts the phase information through the forward and inverse functions. The phase information is 0~π in one and two quadrants and −π~0 in three and four quadrants, and it will jump in two and three quadrants. The value of the phase information $\varphi_{x}$ is constrained to be between (−π~π), which is discontinuous, and it is called the wrapped phase. The wrapped phase of the echo-sampled signal is shown in Figure 14.

Therefore, the phase needs to be subjected to the unwrapping operation. The specific solution algorithm for serializing the wrapped phase is shown in Equation (28).

(24) $$\varphi_{i}=\varphi_{i-1}+W_{i}$$

where $W_{i}$ defines the unwrapping operator:

(25) $$W_{i}=\left\{\begin{array}{c} \varphi_{i}-\varphi_{i-1}-2\pi \;\;\;\;\;\;\;\;\;\;\;\;\;\;\;\;\;\;\;\;\;\;\;\;\;\;\;\;\;\;\;\;\;\;\;\;\;\;\;\;\;\varphi_{i}-\varphi_{i-1}>\pi \;\\ \varphi_{i}-\varphi_{i-1}+2\pi \;\;\;\;\;\;\;\;\;\;\;\;\;\;\;\;\;\;\;\;\;\;\;\;\;\;\;\;\;\;\;\;\;\;\;\;\;\;\varphi_{i}-\varphi_{i-1}<-\pi \\ \varphi_{i}-\varphi_{i-1}\;\;\;\;\;\;\;\;\;\;\;\;\;\;\;\;\;\;\;\;\;\;\;\;\;\;\;\;\;\;\;\;\;\;\;\;\;\;-\pi <\varphi_{i}-\varphi_{i-1}<\pi \end{array}\right.$$

The period of the ultrasound echo is calculated by the phase difference and time difference of the characteristic positions of the echo, as shown in (30).

(26) $$T_{l}=\frac{2\pi \cdot \left(m_{2}-m_{1}\right)}{\left(\varphi_{m_{2}}-\varphi_{m_{1}}\right)\cdot f_{se}}$$

where $m_{1}$, $m_{2}$ are the points at the characteristic positions, i.e., the two positions corresponding to the zero phase in 19. $\varphi_{m2}$, $\varphi_{m1}$ are the phases corresponding to the unwrapped phase, and $f_{se}$ is the sampling reconstruction frequency. The TOF of the ultrasound echo is $T_{l}$.

## 5. Ultrasonic Thickness Measurement Results and Analysis

### 5.1. Velocity of Sound Calibration and Calculation of TOF

According to the sampling reconstruction technique, it can be seen that the size of the sampling period $T_{s}$ and the excitation period $T_{x}$ are set by the internal system timer; the excitation period is $T_{x}$ = $R_{1}$/144 M. The sampling period is $T_{s}$ = $R_{2}$/144 M. The setting of the sampling period must be greater than 0.41 μs and satisfy the improved equivalent Equation (1), in which it is known that the number of sampling points *n*, the number of sampling periods *m,* and the counter accumulator $R_{1}$ and $R_{2}$ setting values satisfy the following relationship:

(27) $$m\cdot R_{1}=n\cdot R_{2}$$

Three thicknesses of 45 steel-stepped specimens were selected to calculate TOF and calibrate the speed of sound. Thickness measurements were carried out on specimens with calibrated thicknesses of 3.010 mm, 10.003 mm, and 24.047 mm. The calibrated thickness of 3.010 mm was used as an example.

**Figure 15:**
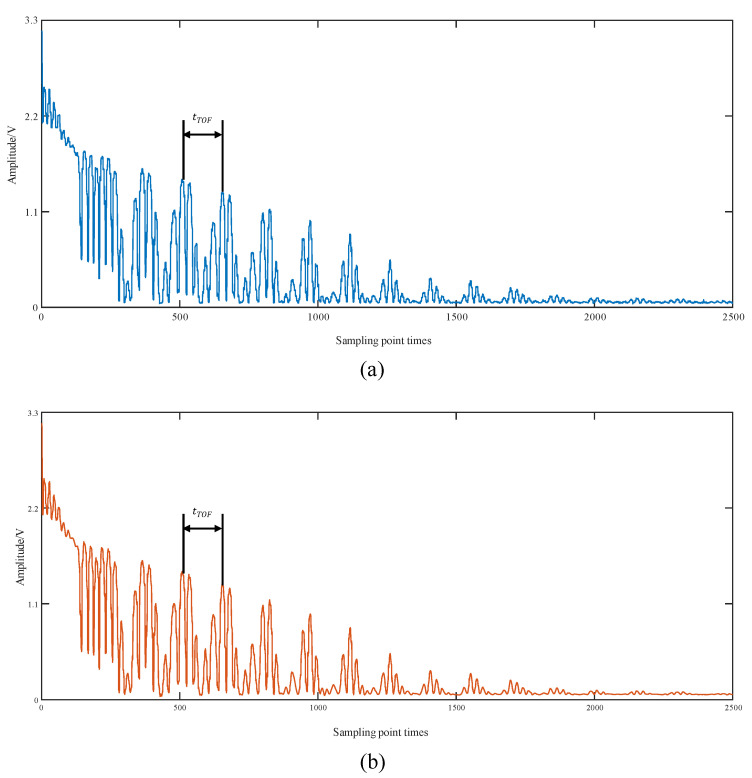
(**a**) Reconstructed ultrasound echo signal (3.010 mm); (**b**) ultrasound signal after wavelet noise reduction (3.010 mm).

The first is to set the parameters $R_{1}=18,000$, $R_{2}=67$, m=67, n=18,000. According to Formula (2), it can be seen that the sampling reconfiguration frequency $f_{se}=144\;MHz$, the sampling frequency is relatively increased by 67 times. ADC sampling of ultrasound echo signals can be realized. Obtaining 67 excitation cycles and 18,000 sampling points. 18,000 points first reconstruct 36,000 points, 2 bytes, 72 k memory. stm32F407 has 128 k memory. The actual data to be processed is the initial 4096 points sampled, and the floating point number occupies 4096 × 4 space, i.e., 16 k data for the FFT transform. Therefore, the memory space for sampling and reconstruction can be multiplexed. Reconstructing the sampled signal via the above signal arrangement Equation (3) to obtain the complete echo signal for one excitation cycle. The sequence diagram of the reconstructed ultrasound echo signal at the intercepted 2500 points is shown in Figure 15a. The reconstructed signal is then subjected to wavelet noise reduction by the multiresolution thresholding method, and the processed signal is shown in Figure 15b.

**Figure 16:**
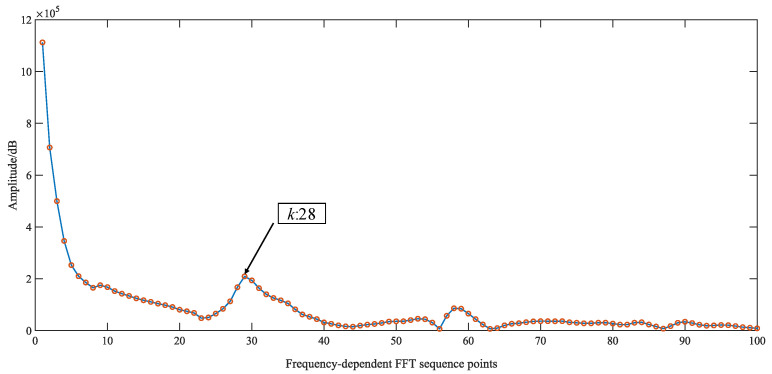
FFT spectrogram of ultrasound signal (3.010 mm).

Extracting the phase information based on the method of shifted sine fitting and calculating the TOF. The FFT roughly estimates the low-frequency harmonics of the echo signal, selecting *N* = 4096 points for the FFT transform. Its spectrogram is shown in Figure 16. The position of the frequency point corresponds to the low-frequency harmonics of the ultrasound echo *k* = 28. The phase interval δ=0.043 and the segment length of the shifted sine fit *L* = 146 were determined according to Equations (20) and (24), respectively.

**Figure 17:**
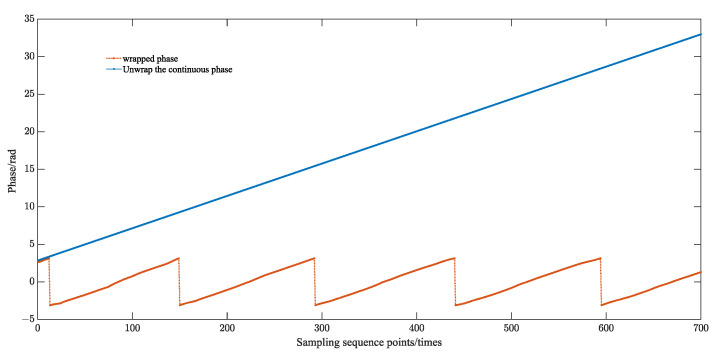
Phase information (3.010 mm).

Using a moving sine fitting algorithm to extract the phase information of the ultrasound echo signal. The phase-out $\varphi_{x}$ corresponding to the echo signal after noise reduction is calculated according to Equations (21) and (22), etc. Solving the parcel Equation (28) to solve the phase. The continuous phase information is shown in Figure 17. Finally, the fitted echo period is calculated by the phase difference Equation (30). That is, the TOF is $t_{TOF}=1.015$ μs.

**Figure 18:**
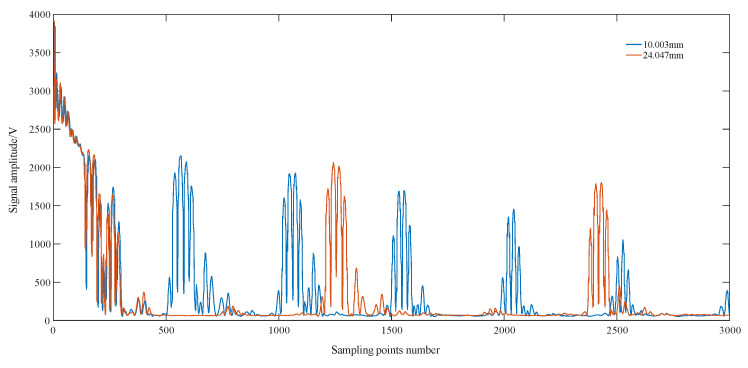
Ultrasound echo signal maps for different thicknesses.

**Figure 19:**
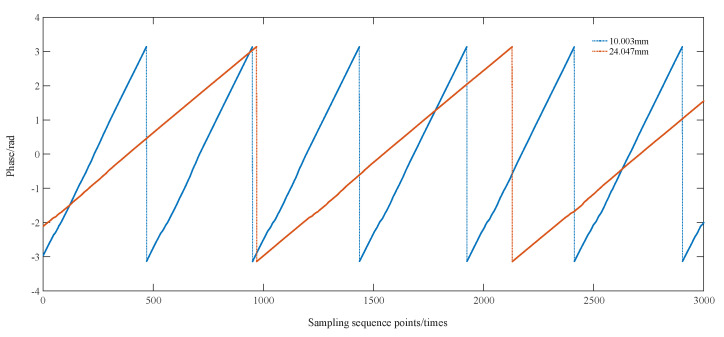
Graph of phase information for different thicknesses.

For the remaining two sets of calibrated thickness values of 10.003 mm and 24.047 mm, the TOF process is the same as above. The ultrasound echo signals for the two thicknesses are shown in Figure 18, and the phase information is shown in Figure 19.

**Figure 20:**
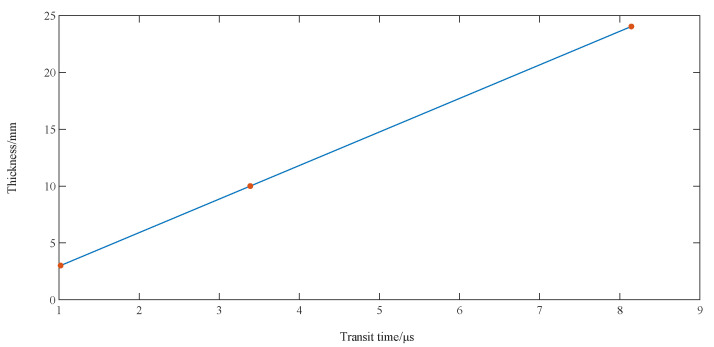
Fitted line graph for sound speed calibration.

The TOF was 3.373 μs and 8.112 μs for the thicknesses of 10.003 mm and 24.047 mm, respectively. A linear fit of the calibrated thickness values to the measured TOF resulted in a straight line for the thickness versus TOF relationship, with the slope of the line being one-half of the calibrated speed of sound, as shown in Figure 20.

The linear regression model is given by:

(28) y=Ax+B

where *y* is the calibrated thickness measurement sequence. *x* is the transition time sequence. *B* is the zero calibration value. *A* is the parameter that represents the sound–velocity relationship. The expression for the speed of sound is:

(29) $$c=2A=2\frac{n\sum x_{i}y_{i}-\sum x_{i}\sum y_{i}}{n\sum {x_{i}}^{2}-{\left(\sum x_{i}\right)}^{2}}$$

Equations (32) and (33) give a calibrated speed of sound of 5928 m/s for 45 steel and a zero calibration value of 0.0029 mm.

### 5.2. Determination of Thickness Measurement Range

**Figure 21:**
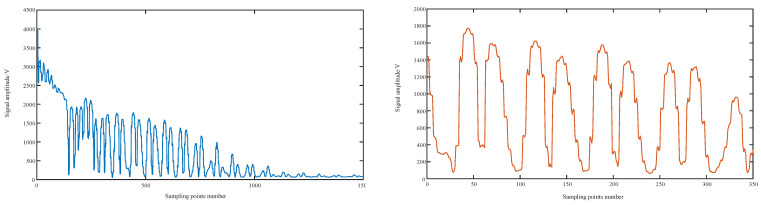
Signal obfuscation and echo characteristic signal extraction plot.

The selected ultrasonic thickness test block range is from 1 mm to 50 mm, the selected ultrasonic probe has a fixed frequency of 2.5 MHz, the minimum time interval between the transmitted signal and the echo signal is *t* = 0.8 μs, the calibrated speed of sound in 45 steel is 5928 m/s, and the minimum thickness of the measurement is $d_{m}$= 2.37 mm. However, the method applied in this experiment is to select the characteristic positions of multiple echo signals in a finite attenuation signal and to find the phase difference, which can remove the confusing overlapping signals and extract the useful echo characteristic signal. As shown in Figure 21, the minimum thickness of a 1 mm specimen can be measured. According to the system’s ultrasonic excitation period of 125 μs, the conditions of signal attenuation, and the theoretical maximum measurement thickness of 150 mm, the system’s thickness measurement range meets the requirements of 1 mm to 50 mm.

### 5.3. Thickness Measurement Results

**Figure 22:**
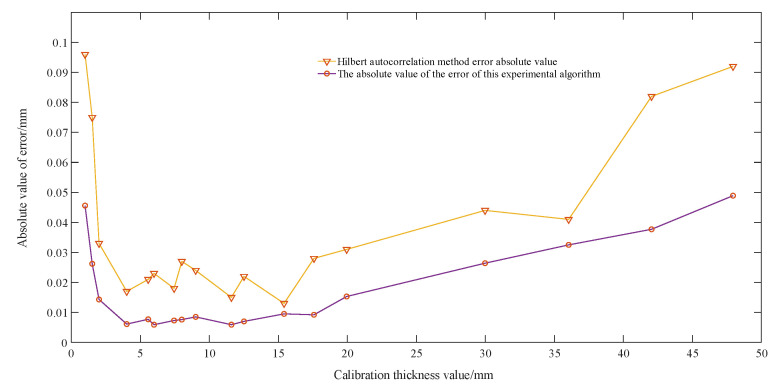
This experimental algorithm and the Hilbert autocorrelation method measure the absolute value of the error in steel.

The remaining thickness of the ultrasonic step test block was measured using this experimental algorithm. The crossing time was recorded. The measured thickness was calculated using the calibrated sound velocity of 5928 m/s with a zero calibration value of 0.0029 mm. And the ultrasonic thickness measurement block comparison experiment was performed by the Hilbert autocorrelation method [13]. The measured thickness results are shown in Table 2, and the comparison graph of the absolute value of error is shown in Figure 22.

Based on the above data, it can be seen that the error of the algorithm for phase feature extraction in this paper is less than that of the Hilbert autocorrelation method. In this paper, the consumption of systematic measurement time is mainly reflected in the following aspects, as shown in Table 3.

The system has a fast measurement speed with a single measurement time of about 40 ms.

The Hilbert autocorrelation method mainly performs the Hilbert transform on the self-multiplying echo signal first, and then undergoes the autocorrelation method to obtain TOF. The Hilbert transform is first obtained by an *N*-point FFT operation to obtain the spectrum. The positive half of the spectrum is multiplied by −*i*, the negative half by *i*, and the DC component is set to 0. Finally, the frequency domain signal is inverted into a time domain signal by *N* FFTs to complete the Hilbert transform. The Hilbert transform is completed by multiplying the frequency domain signal by *N +* 3/2*Nlog*2*N* times and adding the complex numbers by *N +* 3*Nlog*2*N* times. The traditional autocorrelation algorithm is computationally intensive and requires $N^{2}$ multiplications and *N(N* − 1) additions. Now the autocorrelation operation is generally converted from the time domain to a frequency operation. First, the FFT operation is performed on the signal at *N* points. Then the N-point complex self-multiplication is performed. Finally, the FFT is inverted to get the result. It also requires *N +* 3/2*Nlog*2*N* times complex multiplication and *N*+3*Nlog*2*N* times complex addition. Therefore, the Hilbert autocorrelation method requires a total of 2*N* + 3*Nlog*2*N* complex multiplications and 2*N* + 6*Nlog*2*N* complex additions.

**Figure 23:**
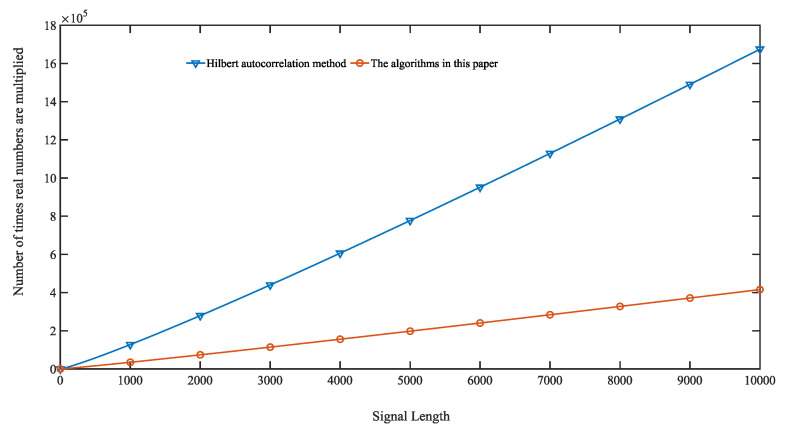
Plot of computation versus signal length.

The complexity of the algorithm in this paper is mainly in the FFT operation and the shifted sine fitting operation. It requires one FFT operation with *N* points, 1/2*Nlog*2*N* complex multiplications, and *Nlog*2*N* complex additions. The shifted fit requires only 15 real multiplications and 20 real additions per recursion. One complex multiplication requires four real multiplications and two real additions. The Hilbert autocorrelation method requires 8*N* + 12*Nlog*2*N* real multiplications. The algorithm in this paper requires 15*N* + 2*Nlog*2*N* real multiplications. Its real number multiplication complexity simulation diagram is shown in Figure 23.

Figure 23 reflects that the algorithm in this paper has less complexity than the Hilbert autocorrelation method, and the longer the length of the ultrasound echo signal to be measured, the better the performance.

## 6. Conclusions

Aiming at the limitations of high-frequency signals needing a high-frequency sampler and the difficulty of accurate extraction of echo signal features. This paper first proposes an improved sampling reconstruction method, a moving sine fitting method to accurately extract the phase information of echo features, establishes an ultrasonic echo model, and analyzes the performance with the commonly used transit time measurement methods to verify the advantages of the algorithm. The system’s thickness measurement experiments were conducted on different thicknesses of 45 steel calibration and thickness measurement experiments, through the measurement of three sets of thickness of the transit time and its corresponding calibration thickness value to obtain the calibration speed of sound and the zero calibration value of 45 steel. The remaining thicknesses were measured and analyzed for errors. The conclusions show that the measurement error is ±0.01 mm for the thicknesses within 3 mm~20 mm and ±0.05 mm for the other thicknesses within 1 mm~50 mm. The single measurement time is 40 ms. This work is highly integrated and requires only a microcontroller. No additional AD converter chip is needed. The processing method makes the measurement accuracy high and reduces the cost. By setting a reasonable sampling frequency and number of sampling points, a larger range of thickness can be measured. Repeating multiple sampling accumulations improves the signal-to-noise ratio of the echo signal, which has a certain development prospect.

## Figures and Tables

**Table 1:** Results of SNR and RMSE for different thresholding methods.

Threshold Methods	SNR (dB)	RMSE
Fixed threshold	8.72	0.071
Unbiased risk estimation threshold	10.02	0.061
Heuristic threshold	9.70	0.068
The max–min criterion threshold	9.74	0.067
Multiresolution threshold	10.86	0.055

**Table 2:** Thickness measurement results of this experimental algorithm and Hilbert autocorrelation method.

Test Block Number	Calibration Thickness Value/Mm	TOF/μs	The Actual Measured Value of This Experiment/mm	The Algorithmic Measurement Error of This Experiment/mm	Measurements of Hilbert Autocorrelation/mm	Hilbert Autocorrelation Measurement Errors/mm
7A-1	0.994	0.319	0.948	−0.046	1.090	0.096
7A-2	1.512	0.518	1.538	0.026	1.437	−0.075
7A-3	2.007	0.681	2.021	0.014	2.004	0.033
7A-4	4.013	1.351	4.007	−0.006	3.996	−0.017
7C-1	5.568	1.875	5.560	−0.008	5.589	0.021
7A-5	5.992	2.019	5.987	−0.005	5.969	−0.023
7C-2	7.447	2.514	7.454	0.007	7.465	0.018
7A-6	8.004	2.702	8.012	0.008	8.031	0.027
7C-3	9.010	3.036	9.001	−0.009	8.986	−0.024
7C-4	11.598	3.914	11.604	0.006	11.613	0.015
7B-2	12.512	4.218	12.505	−0.007	12.490	−0.022
7C-5	15.415	5.203	15.425	0.010	15.428	0.013
7C-6	17.582	5.934	17.591	0.009	17.610	0.028
7C-7	19.952	6.727	19.942	−0.010	19.921	−0.031
7B-4	29.981	10.123	30.008	0.027	30.025	0.044
7B-5	36.039	12.147	36.006	−0.033	35.998	−0.041
7B-6	42.045	14.197	42.083	0.038	42.127	0.082
7B-7	47.950	16.193	47.999	0.049	48.042	0.092

**Table 3:** System measurement time allocation.

Sampling Reconstruction Response Time/ms	Multi-Resolution Threshold Noise Reduction Response Time/ms	FFT Transform Response Time/ms	Moving Sine Fitting Response Time/ms
8.375	25.26	3.17	1.37

## Data Availability

Data are contained within the article.
